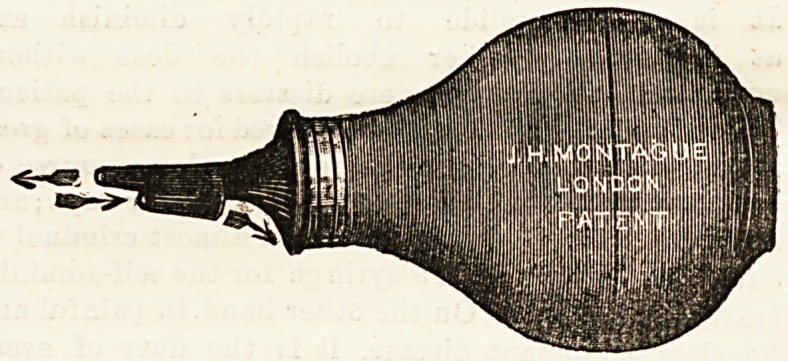# New Appliances and Things Medical

**Published:** 1895-09-14

**Authors:** 


### NEW APPLIANCES AND THINGS MEDICAL.

[We shall be glad to receive, at our Office, 428, Strand, London, W.C., from the manufacturers, specimens of all new preparations and appliances which may be brought out from time to time.]

### FRIEDRICHSHALL WATER, NEW SPRING.

(Proprietors, C. Oppel and Co.; London Agents, The Apollinaris Company.)

In a former notice we expressed the opinion that now the methods of bottling the natural waters had been perfected, it was desirable that the English public should have the opportunity of purchasing the mineral waters of the Continent in this country, and we are glad to be able to call the attention of our readers to this new Friedrichshall water which the Apollinaris Company have recently placed on the market. As an aperient water this new spring is practically identical with the original Friedrichshallbrunnen analysed by Liebig in 1846, and our analyst's figures obtained from the sample submitted to him are closely similar to those of Professor B. Fischer, who analysed the new spring in 1894:—

| | Parts per 1,000. Fischer, 1894. | Our Analysis, 1895. |
|---|---|---|
| Total Chlorine (as chlorides) ... | 8·29 | ... 8·55 |
| ,, ,, (as Magnesium Chloride) | 4·7135 | ... 4·981 |
| ,, ,, (as Sodium Chloride) ... | 7·311 | ... 7·97 |
| ,, Sulphates (as $SO_4$) ... ... | 9·351 | ... 8·11 |
| ,, Carbonates (as $CO_3$) ... ... | 0.2768 | ... 0·287 |

The sulphates in the water are those of soda, magnesia, lime, and potash, the two former being present in about equal quantities, and forming the major part, whilst the carbonate present is nearly entirely that of soda. It will be seen that this composition indicates a characteristically aperient water of not too saline a taste, and one which is of undoubted value.

### TERROL.

(The Terrol Company, 34, Devonshire Road, Forest Hill, S.E.)

Under this fancy name the Terrol Company are introducing a new remedy, called by them the tasteless cod-liver oil substitute, which after a series of expensive and exhaustive experiments they say they have succeeded in producing from crude petroleum. They consider it to be a most valuable therapeutic body, and the only preparation of that article from which all disagreeable features have been entirely removed. From our examination of the sample submitted to us we can confirm this statement as to its tasteless character, and our analyst reports that in his opinion it is a highly purified hydrocarbon allied to petroleum jelly, and having a flashing point of about 420° F. It is free from taste and acidity, and resembles the better class of petroleum jellies, except that its melting point is somewhat lower, as the sample was liquid on arrival. The proprietors quote several testimonials from medical men in this country, who testify to its value as a cod-liver oil substitute, and a member of the profession at Bath says that it appeared to be digested. Although it is well known that the hydrocarbons of petroleum have a value for soothing and allaying cough, we cannot believe that a pure hydrocarbon can be regarded in any sense as a cod-liver oil substitute, and regret that the proprietors have ventured to place this on the market without any physiological evidence in support of their contention. We are quite prepared to admit that as a tasteless petroleum preparation it ranks high, and as such may be of value for internal administration, but until physiological experiments on its actual value as a stimulant to fat absorption are forthcoming, we cannot speak of it as a cod-liver oil substitute. We are aware that Heyerdahl's recent researches on cod-liver oil go far to prove that the value of cod-liver oil in wasting diseases cannot be measured by its relative chemical value as a glyceride; but at the same time, any increase in weight attributed to the use of terrol cannot be due to its digestion, and we hope, seeing that the proprietors say that they have already carried out a series of expensive and exhaustive experiments on its preparation, they will be inclined to continue these experiments from a physiological point of view, and thus establish the true therapeutic value of this new claimant for popular use.

### EXTRACT OF COCOA.

(Teetgen and Co., 52 & 54, Old Kent Road, London, S.E.)

The above manufacturers of cocoas and chocolates have forwarded us a sample of their "Perfect" extract of cocoa. The extract is of excellent quality and highly soluble; moreover it has an excellent flavour. Owing to the purity of preparation this cocoa is one that a physician can safely recommend to his patients. The small percentage of fat present in Teetgen's extract renders it free from the so-called "bilious" properties of many samples on the market. For general household use it is an economical preparation.

### PATENT EAR SYRINGE.

(J. H. Montague, 101, New Bond Street).

This is a most ingenious innovation in the way of ear syringes; it is particularly adapted for domestic use, as it is impossible to do any damage to the more delicate parts of the ear, even in amateur hands. The principle consists in the nozzle of the syringe possessing a "two-way" passage, one for the entering stream of water, and the other for its exit. The nozzle is correspondingly thickened in this

J.H. MONTAGUE LONDON

situation and cannot be passed far into the meatus. Further advantages of this syringe are that the stream of water must pass along the roof of the meatus, and the outflowing stream along the floor; and, secondly, the stream of water as it leaves the ear is directed so as to flow easily into a receiving vessel instead of trickling down the neck as is usually the case when the ear is syringed. The same firm are now supplying the profession with some improved dissecting forceps, nickel-plated, spring-backed, and provided with a guide. The reasonable price of 2s. 6d. should be an additional attraction.

## Figures and Tables

**Figure f1:**
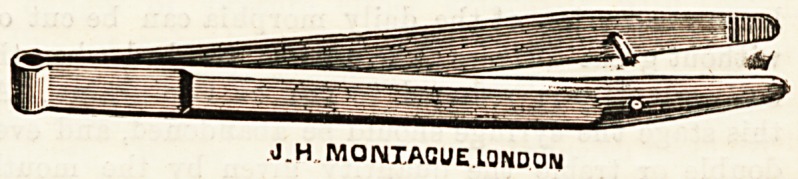


**Figure f2:**